# Descriptive study of patients admitted to a Psychiatric Home Hospitalization Unit in Santa Coloma de Gramenet and Badalona

**DOI:** 10.1192/j.eurpsy.2024.1190

**Published:** 2024-08-27

**Authors:** J. Marti Bonany, D. Garcia Hernandez, D. Tolosa Merlos, R. Romar Navia, R. B. Sauras Quetcuti, M. J. Ambros Ghisilieri, G. A. Mateu Codina, D. Garcia Fuentes, A. M. Coratu, G. De Iturbe Catania, R. Sanchez Gonzalez, M. T. Campillo Sanz, A. Riera Soler

**Affiliations:** Salut Mental Institut, Hospital del Mar, Barcelona, Spain

## Abstract

**Introduction:**

Hospital at home for psychiatric patients is a new emerging resource of delivering acute mental health care in the community. The main objective of this program is to provide intense care to patients with severe mental disorders at home as an alternative to acute admission.

Although home hospitalisation has begun to develop widely in recent years there is a notable lack of studies

The CAEM Psychiatric Home Hospitalization Unit (HAD-CAEM) has been operating since 2018 and takes place in Santa Coloma de Gramenet; and from March 2022 also in a part of Badalona. Both are sociodemographically depressed areas near Barcelona.

**Objectives:**

The aim of this study is to describe the characteristics of patients attended at the Psychiatric Home Hospitalization Unit of our hospital and to study differences according to area and place of referral.

**Methods:**

Socio-demographic and clinical data were collected retrospectively at admission and discharge of all patients treated at HAD-CAEM between March 2022 to february 2023.

Statistical analysis was performed by using SPSS program.

**Results:**

85 patients were included in the study. 45.9% were women. The mean age was 45.5 years (SD 15.58 years). The main diagnoses of the sample were psychosis and schizophrenia (38.8%), Bipolar disorder (23.53%), Depressive disorder (21.18%), schizoaffective disorder (8.24%) and others (8,24%).

54 (63.53%) patients were from Santa Coloma area and 35 (41.18%) from Badalona area.

The total mean duration of admission was 40.22 days (SD 26.18 days), with a mean follow-up of 10.09 visits (SD 5.39 visits) and 2.41 teleassistence (SD 2.62).

The mean duration of admission for Santa Coloma patients was 43.98 days (SD 28.59), and for Badalona patients 33.68 days (SD 20.13). Trend without significance is observed (t= 1.77, p=0.08)

We found differences in the mean duration of admission according to referral location. Acute psychiatric unit 33.25 days (SD 18.06), Mental health Center 51.93 days (SD 33.45), Emergencies 34.28 days (SD 19.69) (F=5.1, p=0.008).

**Conclusions:**

Sociodemografic and clinical característics obtained in our study are consistent with those reported in previous studies. The duration of admission of patients referred from the mental health center is longer than those referred from the acute psychiatric or emergency unit. Home hospitalization teams have been increasing in recent years, being an alternative to traditional hospitalization.

**Disclosure of Interest:**

None Declared

